# From Data to Narratives: Scrutinising the Spatial Dimensions of Social and Cultural Phenomena Through Lenses of Interactive Web Mapping

**DOI:** 10.1007/s41651-022-00117-x

**Published:** 2022-06-16

**Authors:** Tian Lan, Oliver O’Brien, James Cheshire, Alex Singleton, Paul Longley

**Affiliations:** 1 Department of Geography, University College London, London, UK; 2 Department of Geography and Planning, University of Liverpool, Liverpool, UK

**Keywords:** Narrative cartography, Social mapping, Interactive web mapping, Britain

## Abstract

Modern web mapping techniques have enhanced the storytelling capability of cartography. In this paper, we present our recent development of a web mapping facility that can be used to extract interesting stories and unique insights from a diverse range of socio-economic and demographic variables and indicators, derived from a variety of datasets. We then use three curated narratives to show that online maps are effective ways of interactive storytelling and visualisation, which allow users to tailor their own story maps. We discuss the reasons for the revival of the recent attention to narrative mapping and conclude that our interactive web mapping facility powered by data assets can be employed as an accessible and powerful toolkit, to identify geographic patterns of various social and economic phenomena by social scientists, journalists, policymakers, and the public.

## Introduction

Many social and cultural phenomena are often developed within certain geographic contexts or neighbourhoods, which exhibit very clear spatial patterns, for instance, ethnic ghettos or enclaves (Johnston et al. 2002) spotted in many multicultural cities due to the uneven geographic distributions of ethnic communities across different urban settings (Lan et al. 2021). However, the spatial logic of social and cultural landscapes is sometimes not explicit until they are superimposed on various maps showing the spatial context of the events, using a variety of cartographic representations and techniques such as dot maps, contour maps, flow maps, choropleth maps and mashups (e.g., Gibin et al. 2008; O'Brien and Cheshire 2016; van Dijk and Longley 2020). The stories, narratives, and patterns of social and cultural phenomena along spatial dimensions are more pronounced when they are geo-visualised using maps and cartographic design as a way of visual storytelling (Roth 2021).

Maps are abstract and simplified models of different facets of the real world. The relationship between maps and storytelling can be viewed from a cartographic perspective that maps are used to display the spatial structure of the latter and maps have the narrative power (Caquard and Cartwright 2014). Maps have long been employed as natural languages of spatial storytelling by geographers and other scientists (See, for example, Kraak and Kveladze 2017; Kwan 2008; Mocnik and Fairbairn 2018; Segel and Heer 2010; Wood 1987). New forms of spatial expressions about places and stories associated with places are also witnessed in the literature of film studies, visual arts and digital humanities (Caquard 2011). Amongst the narrative cartography community, the use of map-based storytelling along with geo-awareness and spatial literacy have also been promoted in the context of citizen science (Kerski 2015). Story-based mapping is a very intuitive and informative way of visual presentation in data journalism’s storytelling toolsets as well. For example, the frequently updated COVID case map of the USA showing publicly debating topics such as the latest hot spots of infections and vaccination rates, produced by the New York Times magazine (https://www.nytimes.com/interactive/2021/us/covid-cases.html).

That being said, social and cultural mapping is not new and there is a long history of using maps to investigate social issues centuries ago, for example, the topics on health, poverty and identity (Vaughan 2018). Social reformer Booth (1893) visualised the findings from his painstaking inquiry of urban poverty in late Victorian London in a series of street maps colour coded by the social status of the residents, known as the Booth Poverty Maps (see https://booth.lse.ac.uk/map). Almost simultaneously on the other side of the Atlantic Ocean, similar concepts of colour coded, highly granular social mapping were employed to represent the nationality of households in the Hull House Map in contemporary America (https://digital.library.cornell.edu/catalog/ss:3293796). The striking urban poverty and residential segregation narratives conveyed from the pioneering work prove the efficacy of narrative cartography in mapping the landscapes of society and culture.

In recent years, social and cultural mapping applications thrive in cyberspace beyond these historical traditions and links, with the proliferation of the web GIS technologies and various more readily available traditional and novel data sources including census population data, governmental registration records, consumer data, and other data. New big data assets such as consumer data (Longley et al. 2018), which are typically richer in socio-economic attributes and more frequently collected than conventional statistical sources, are more appropriate for developing narratives. Smith (2016) provides a review of online interactive thematic mapping applications and summarises the potential of web mapping techniques for socio-economic research. Gibin et al. (2008) have developed the Google Map mashup based LondonProfiler, which facilitates online exploratory cartographic visualisation in a range of policy concerns and inspired later web mapping facility development including DataShine, CDRC Maps and CDRC Mapmaker in this paper. O'Brien and Cheshire (2016) have created the DataShine web mapping platform to visualise a list of socio-economic indicators drawn from various open demographic data. Van Dijk and Longley (2020) have used a series of truncated kernel density estimator maps to display snapshots of surname distributions in Britain at different time points. Lan and Longley (2021) have devised a web mapping portal to create geodemographic profiles for standard or bespoke regions and areas. Romano and Hedley (2021) have developed a Virtual Reality and Mixed Reality enhanced web mapping system to connect audiences in narratives, experiences or emotions of past events and places.

Although web mapping makes various interesting social and cultural geographies and narratives more accessible for wider map user communities, many current applications have several limitations in terms of interactivity, analytical power and reporting functionalities. Most of the web mapping applications are ‘slippy’ maps, which only provide very basic operations (e.g., pan or zoom). They fall short of more user-oriented interactions. Sometimes, web mapping users just want to focus on their own areas of interest, to show or hide places based on attribute filtration, and to export to or reference maps in their storyboards or reports. Moreover, some web mapping facilities are self-hosted and pre-rendered raster tiles of simple choropleth maps, which might be misleading in some cases—for example, a false impression of high population density in urban parks just because these parks are within the boundaries of the mapping units. From a technical point of view, millions of self-hosted image tiles require high internet traffic and high maintenance due to the potential concurrency of visits. However, as the modern web mapping tool stack has evolved, it also opens up opportunities to cope with these limitations.

In this paper, we present our recent development of a web mapping portal in the Consumer Data Research Centre (CDRC, https://www.cdrc.ac.uk/), the CDRC Mapmaker (https://mapmaker.cdrc.ac.uk/), focusing on the narrative functionalities and improvements with respect to the above limitations. We draw upon a wide variety of socio-economic and demographic data packs and umbrella the thematic maps such as health asset accessibility, deprivation indices, housing price, and the UK Output Area Classification under five broad topics—Population and Mobility, Retail Futures, Finance and Economy, Digital, and Transport and Movement. We purposely leave the data hosting and web GIS creation to third-party web mapping services and focus on the map design, configuration, and data presentation. We introduce the urban/buildings mask layer to tackle the issue of unpopulated areas within choropleth maps. We also enable the analytical and reporting features such as filtering areas and places by attributes and exporting maps and links to PDF reports or storyboards. We further explain and elaborate on these improvements and new features in the next section as well as in the case study narrations.

## CDRC Mapmaker and Interactive Web Mapping

### Datasets

CDRC Mapmaker has been developed as a map visualisation and analysis online tool which shows the various consumer-related datasets from the CDRC Data service, and some other UK socioeconomic datasets. One important data asset at CDRC is the Linked Consumer Registers (Lansley et al. 2019), which contain c. 885 million records in total compiled from the public version of electoral rolls and other consumer sources. They provide annual snapshots of the near-complete adult population in the UK from 1997 to 2021 and have been used in three Mapmaker thematic maps such as Ethnicity Estimator, Residential Mobility Index and Residential Moves: Distance/Deprivation. Other datasets underpinning the Mapmaker mapping system include useful large open demographic data derived from official statistics or research outputs such as the Output Area Classification and Deprivation indices.

### CDRC Mapmaker

**Fig. 1:**
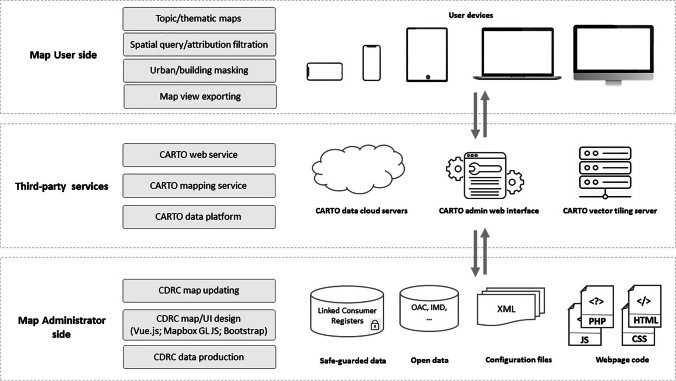
An illustration of the Mapmaker web mapping system

Mapmaker web mapping facility (See the illustration in Fig. 1) is a replacement for CDRC Maps, a simple “slippy” mapping website, and moves the visualisation and storytelling elements of the CDRC outputs forward in several ways. Firstly, it presents the mapped data using the latest web technologies and standards such as responsive web design and vector tiling. The website is fully responsive, that is, adaptive to viewing on different sizes of devices, reflecting the recent trend towards viewing complex websites with smartphones and tablets with smaller but higher resolution screens than the traditional desktop web browsing experience. The website is built on the Node and Vue JavaScript frameworks, both of which are widely used in web design and development. The site is assembled using Node Package Manager (NPM). This allows for easy integration of standard components, meaning that the amount of specific code to create the website itself is relatively small. Vector tiles are used to display the map layers, which are normally smaller in size compared to the traditional raster tiles covering the same geographic area. This reduces the data traffic transferred from map servers. These tiles are rendered in parallel on-the-fly in vector format in users’ web browsers using Mapbox GL JS.

Secondly, the data presented on the website is entirely database-driven, which substantially cuts down on the resources required to host and maintain the website, and makes updates and additions relatively simple. Previously, a huge number of raster image “tile” files needed to be pre-generated and stored on a server—any change to the data, such as additional rounding required due to updated disclosure control requirements—would require time to regenerate the images. In Mapmaker, the vector tiles are rendered in real-time according to specified configuring rules. With the new architecture, updating the database takes immediate effect on the mapping platform. This could be simply via a download, modification and re-upload of a set of shapefiles and corresponding configuration files using the CARTO web interface.

Finally, it is not dependent on any of CDRC’s own infrastructure. The code itself is stored on GitHub, the production website is on a third-party web host, and the data is on the CARTO data platform. This allows CDRC to focus on the production of new datasets and research support to others using the data and mapping, rather than needing to provide and maintain hardware to support the service. This also means Mapmaker can manage visitor spikes due to media interest and other events—the data platform being a cloud service means it can be scaled up efficiently to service multiple users, and this can be achieved automatically by CARTO themselves. The website itself is a small, lightweight download and once it is loaded to each user’s browser, no additional download from the website is required—the downloaded code itself retrieves the data directly from the CARTO data platform.

CDRC Mapmaker presents two kinds of maps—geodemographic classifications, and metrics. Geodemographic classifications are nominal variables and are typically shown with very different colour hues in order to emphasise the difference between each category. They can be hierarchical, with each category having one or more levels of subcategories. On the other hand, metric maps show a single numerical variable about the dataset so typically use a colour ramp that diverges between two colours or has a single colour hue, to show the gradual change in the value across areas. In the data platform, the actual value is typically stored, and the Mapmaker website’s configuration splits these into banded categories and assigned the appropriate colour.

All maps presented on CDRC Mapmaker, both classification and metric maps, are displayed in the same way—with a full-screen slippy map augmented by a small key and control panel. Building, urban footprint, labels, contextual information and administrative boundary layers can be toggled on and off, and postcodes can be used to zoom into an area. Mousing over an area containing data from the currently selected map may show attribute information while clicking on such an area will typically show additional information such as pen portraits (for classifications), further metadata and context, or the population-weighted proportion of the current administrative area (or custom-drawn area) that contains the current category.

## Case Study Narratives Using Mapmaker

We next use three pieces of narratives—neighbourhood deprivation, internet usage, and residential movements and ethnicity—as case studies to demonstrate how web mapping can be used for interactive storytelling. Although only static screenshots of the maps are shown here, readers are encouraged to explore the full set of interactive maps and stories with embedded interactive maps (e.g. https://data.cdrc.ac.uk/stories/iuc). Relevant maps have one or more “story” web links shown on the side panel of the map. These link to long-form text articles on a complimentary website which may contain screenshots or embedded versions of the maps at relevant points in the text, to lead a reader through a step-by-step narrative related to the maps’ data.

### Neighbourhood Deprivation and Inequalities

Inequalities, examined along different dimensions such as income, gender, ethnicity, health, and age, are the most debated topics of social investigations in the UK, which lead to unequal life chances and outcomes. For example, the UK Office for National Statistics reveals the uneven distribution of household wealth—the top decile of households owned 45% of total aggregate household wealth in the UK (Office for National Statistics 2015). Among many contributing factors, there are marked regional disparities and geographies of socio-economic inequalities in the UK observed as “coldspots” or “left-behind” areas (Local Trust & Oxford Consultants for Social Inclusion 2019), as well as the enduring “north–south divide” (Longley et al. 2021). The Index of Multiple Deprivation (MHCLG 2019) provides a summary measure of relative deprivation for the Lower Layer Super Output Area (LSOA, a UK Census release geography with an approximately 1500 population in England and Wales) in England across a spectrum of 7 weighted Domains: Income, Employment, Health Deprivation and Disability, Education, Crime, Barriers to Housing and Services, and Living Environment. Since geography plays a crucial part in the neighbourhood deprivation and inequalities in the UK, it is intuitive to represent the regional and local disparities by using web-based online maps. The CDRC Mapmaker has two maps related to this theme: Deprivation Indices (https://mapmaker.cdrc.ac.uk/#/index-of-multiple-deprivation) and Deprivation Rank Change (https://mapmaker.cdrc.ac.uk/#/deprivation-change).

**Fig. 2:**
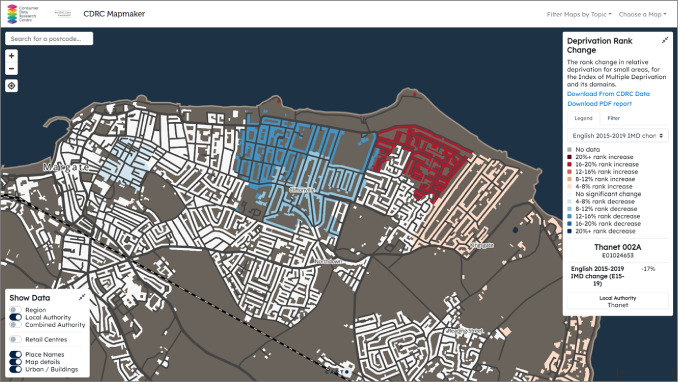
The contrasting deprivation changes in Cliftonville, Margate, 2015–2019

Changes in deprivation rank over time are interesting indicators to explore, although they should never be compared on absolute scales. Moreover, with caveats such as possible calculation methodology and small area boundary changes, we take the Deprivation Rank Change map as a case study here to investigate the relative performance and changes of areas. We pan the map and zoom into a coastal town, Margate, on England’s southeast coast to illustrate the neighbourhood changes from 2015 to 2019 (see Fig. 2). Margate was historically a very popular seaside town but now is one of the most deprived places in the UK, although it still attracts a lot of holidaymakers from London. Figure 2 shows the rank changes of Cliftonville in the Thanet District of east Margate. Compared to traditional choropleth maps, our map foregrounds the building footprints and street networks and downplays the unpopulated areas such as the parks, farmlands, and beaches in dark grey on the map.

We also spot a stark contrast in relative deprivation rank changes 2015–2019 between the west (in blue) and east (in red) sides of Princess Margate Avenue. The eastern part of Cliftonville is reported to have about a 17% deprivation rank increase, which suggests this part has become more deprived relative to other areas in England during the four years of development; while on the other side of the street, the story is completely different—the western part relatively outperforms its counterparts by a 13% decrease in the rank. To track down the factors that affect the overall deprivation scores, further investigations can be achieved by using the dropdown menu on the right legend panel and visualising the indices from the 7 Domains separately. In so doing, we find that there is no significant change in the Environment Domain in both parts and that they both become more deprived in the Housing Domain and performed relatively well in the Crime Domain. However, the Income, Employment, Education and Health Domains make the two neighbouring areas end up with completely different performances with respect to the relative change in the overall deprivation rank. Therefore, when making localised revitalisation and levelling-up policies for these “left-behind” areas, considerations of improving the underperformed Domains should be prioritised.

Further investigations of neighbourhood changes can be conducted in combination with two other Mapmaker themes: the annual updated Ethnicity Estimator modelled from the Linked Consumer Registers (Lansley et al. 2019) and the Temporal Output Area Classification (TOAC). Besides, an “Urban/Buildings” toggle button is enabled in the layer control panel on the bottom left corner for users to switch between a standard choropleth map and a street/building based map. The underpinning data for making the map can be downloaded via the hyperlink in the right-side panel to the CDRC data service. In addition to that, the map can also be exported conveniently to a PDF report using the hyperlink in the panel.

### Internet Usage and Digital Exclusion

Various emerging information and communications technologies have profoundly changed society and people’s lifestyles during the past decades, as netizens move part of their daily activities into the digital world and cyberspace—for example, online shopping, remote learning, video conferencing, online social network and so forth. The Covid-19 pandemic and lockdowns further thrust this trend due to different social distancing measures. However, it should never be overlooked that there are still many left-behind or even excluded members of the society in the wave of the digital revolution for a variety of economic and demographic reasons (Longley and Singleton 2009). They might experience disadvantages or limited access to these resources, digital skills and online information resulting from learning barriers, physical disabilities, and availability or affordability of IT equipment and broadband internet.

In response to the issue of digital divide and exclusion, the CDRC Mapmaker displays two sets of maps related to this theme: Broadband Speed and Availability (https://mapmaker.cdrc.ac.uk/#/broadband-speed) and Internet User Classification (https://mapmaker.cdrc.ac.uk/#/internet-user-classification). The broadband speed data are annual average wired internet speed by output area for both residential and commercial addresses published by Ofcom. The Internet User Classification, developed by Alexiou and Singleton (2018), is a demographic neighbourhood classification of 10 distinctive supergroups, ranging from “e-Cultural Creators” to “e-Withdrawn.” The classification provides insights into how people in different small areas in the UK engage with internet usage and online activities, which is based on input information from a variety of sources including the UK census population data, Oxford Internet Institute survey, and internet infrastructure data from Ofcom.

**Fig. 3:**
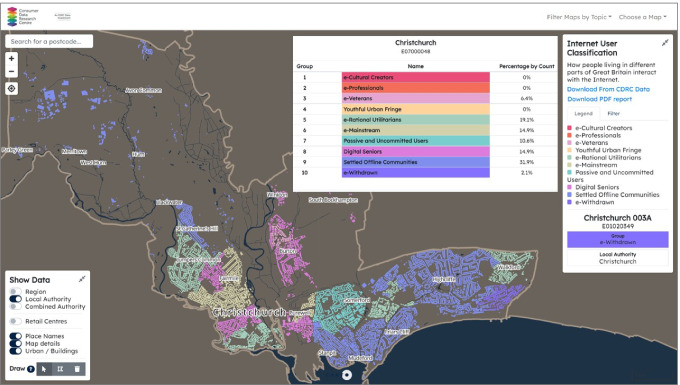
Internet User Classification filtered by Christchurch District, also showing the map legend of the colour codes and the percentages of LSOAs belonging to each of the 10 Groups

**Fig. 4:**
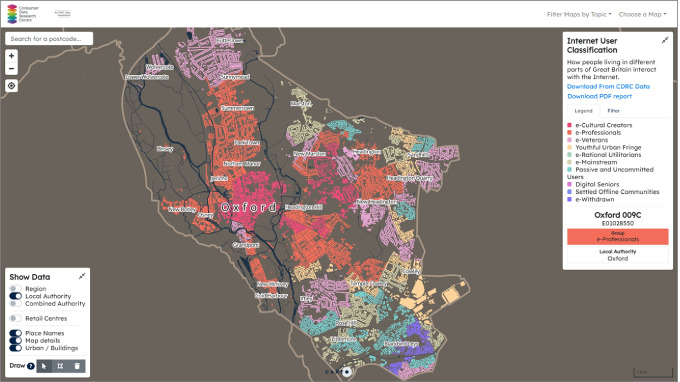
Internet User Classification filtered by Oxford District showing the map legend of the colour codes

Through the map visualisation of the Internet User Classification in Mapmaker, we have identified several Local Authority Districts across the UK, which have significantly higher proportions of communities that are least engaged with the internet. Most of these districts are small coastal towns with notably larger shares of senior White British residents than the national average, which are also known as the “Silver Towns” (Lan and Longley 2021) such as Blackpool, Southend, Swansea, and Christchurch. Figure 3 takes Christchurch as an example and shows the geographic distribution of the Internet User Classification groups as well as the proportions of the LSOAs falling within each of the 10 Groups. The Settled Offline Communities Group is the modal class of the neighbourhoods, which accounts for 31.9% of the LSOAs in this district. The pen portrait of Settled Offline Communities describes members of the Group as elderly, retired White British who might have only limited or indeed no engagement with the internet. Their online activities are more likely to be conducted via computers rather than mobile devices and are perhaps restricted to information seeking and limited online shopping rather than social networking or gaming (Alexiou and Singleton 2018). In contrast, Fig. 4 displays the Classification in Oxford dominated by e-Professionals (33.3%) where the high-tech industries and motor manufacturing companies are. At the core of the city lie the Colleges of the University of Oxford, which are mostly classified as e-Cultural Creators that have high levels of Internet engagement, particularly regarding social networks, communication, streaming, and gaming.

### Residential Movement and Ethnicity

Both intra- and inter-city residential movements of households provide important population dynamics to spatial and social mobility, resulting in various neighbourhood outcomes such as ethnic residential segregation (Clark and Fossett 2008). However, residential mobility and ethnic residential segregation–related studies were previously limited by the coarse temporal and spatial granularity of data sources until the recent proliferation of longitudinal population data (Coulter et al. 2016; Lan et al. 2021). The Linked Consumer Registers (Lansley et al. 2019) are among the novel data assets that have been used to study residential mobility (van Dijk et al. 2021) and segregation (Lan et al. 2021) in British society.

**Fig. 5:**
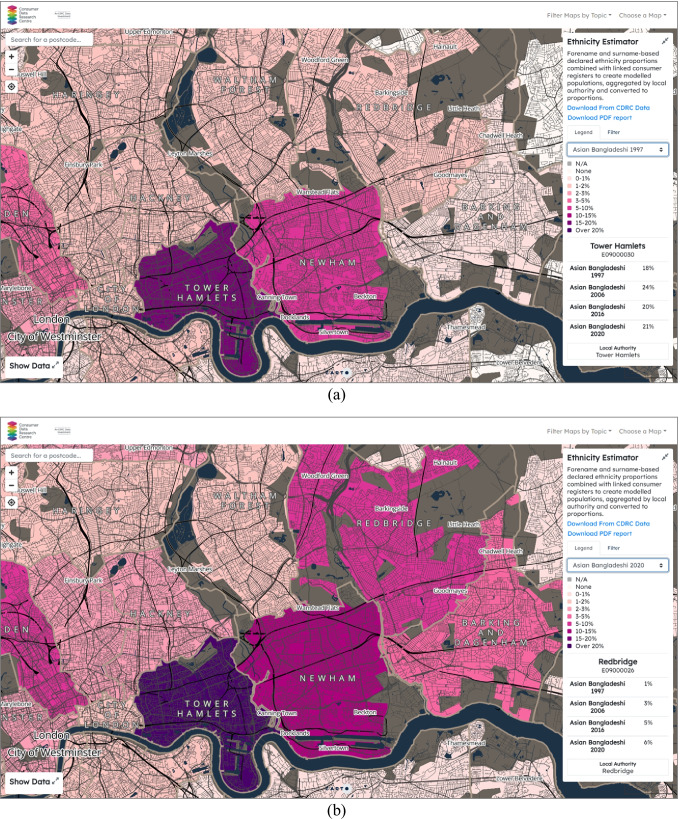
Proportions of the Bangladeshi population by the London Boroughs in central and eastern London: (**a**) 1997, (**b**) 2020

Making use of the georeferenced addresses and modelled ethnicity from names (Kandt and Longley 2018) in the Linked Consumer Registers, the Mapmaker presents a series of proportion maps by the census ethnic groups in the UK in the four-time points—1997, 2006, 2016 and 2020 (https://mapmaker.cdrc.ac.uk/#/modelled-ethnicity-proportions). This allows researchers, policymakers, and the public to examine the geographic distributions of specific ethnic groups overtime at the local authority level. Figure 5(a) and (b) shows the changes in the Bangladeshi communities in London by comparing the proportions and distributions of the populations over the decades. Unlike other minority groups, most Bangladeshi communities are reported to concentrate in London (Lan et al. 2021), particularly in the London Borough of Tower Hamlets coloured in dark purple in Fig. 5(a) where almost 20% of its residents in 1997 were Bangladeshis. By hovering a mouse pointer over Tower Hamlets on the map, the information in the right-side panel also indicates the proportions of the Bangladeshi residents in this Borough increased from 18% in 1997 to 21% in 2020.

Apart from Tower Hamlets, the neighbouring Borough Newham and Camden also recorded high percentages of Bangladeshi residents in 1997. In 2020, a considerable increase in the proportions of Bangladeshis has been spotted in London Boroughs further beyond the previous concentrations in Tower Hamlets and Newham in Fig. 5(b), such as Redbridge and Barking. During the recent two decades, there have been urban gentrification and regeneration projects completed in the neighbourhoods in Tower Hamlets, for instance, Shoreditch, Spitalfields, Banglatown, and Whitechapel. East Village in Stratford, developed as the 2012 London Olympic sites, has been regenerated and adapted to create new residential buildings, shopping malls and restaurants. The expansion of the Bangladeshi communities beyond the East End is likely to be connected with the residential moves and displacement of many deprived residents from the city centre (van Dijk et al. 2021) resulting from these urban redevelopment projects. However, it is worth noting that the underpinning data have the potential to scrutinise social and demographic changes since the 2011 Census at finer granularities, for example, at the neighbourhood or even household level, although they are aggregated to local authority districts in the map for disclosure control purposes.

## Conclusions

Here we introduce an interactive web mapping platform that facilitates the narratives and storytelling of different social and cultural phenomena. Using the three representative case studies, we demonstrate how various open social and demographic data (e.g. the Linked Consumer Registers) can be employed to investigate pressing social issues and make stories out of maps. We adopt state-of-the-art web mapping techniques such as vector tiling and put forward advanced features including the urban/building mask layer, attribute filtration, and map view exporting functions, which help users to generate map stories of their own interests. The interactive online maps are proved to be effective and informative ways of presenting the spatiotemporal sequences of stories.

Narrative cartography is not a concept that has just emerged recently. Instead, early social investigation pioneers such as Charles Booth had already started to use cartographic representations to communicate findings and results of their social inquiries and investigations when maps were still available mostly in paper forms. The revival of the recent interest in narrative mapping is attributed to several reasons. First, the resurgence of interest results from the thriving of an interdisciplinary research community that aims to promote computational social science (Lazer et al. 2009) and spatially integrated social science and humanities (Sui 2010). Geo-visualisation or mapping becomes a very powerful analytical toolkit for researchers in social science and humanity domains. Second, various new data sources emerge in the past decades and become more and more accessible. Amongst these, many data are either explicitly or implicitly associated with spatial and locational attributes that can be mapped and analysed using GIS. Finally, mapping techniques have been advanced considerably since the digital age, for instance, the advancements introduced in the “CDRC Mapmaker and Interactive Web Mapping” section, including web GIS, vector tiling, and so on.

Narrative mapping matters, as it is not only about telling eye-catching stories but also, more importantly, helps to pinpoint what and where various social issues are. For example, these deprived neighbourhoods, left-behind communities, digitally excluded populations and socially, economically squeezed ethnic minorities in the three case studies. It helps us to identify where these hot/cold spots and “left-behind” areas are and suggest possible cures of “levelling up”. However, narrative mapping also comes with caveats. Data sometimes lie and so do maps. Maps could be misleading on occasions due to either biased data or inappropriate cartographic representations, which calls for more attention to be paid to the issues of data provenance and uncertainty underpinning representations and maps. This can be partly fixed by providing mapping data along with maps for reproducibility purposes, which has already been implemented in Mapmaker as introduced earlier. With these caveats noted, interactive web mapping can be used effectively to extract interesting and insightful narratives and patterns out of a variety of open and big data sources about various social and cultural phenomena.

## Declarations

The authors were compliant with the ethical standards.
